# Pancreatic serous cystic neoplasms with spontaneous hemorrhage in a young woman: A case report

**DOI:** 10.1016/j.ijscr.2024.109309

**Published:** 2024-01-28

**Authors:** Toshinao Suzuki, Takahiro Sugiura, Junko Okazaki, Akira Okaniwa, Yu Yoshida

**Affiliations:** a Department of Anesthesiology, Kimitsu Chuo Hospital, 1010 Sakurai, Kisarazu, Chiba 292-8535, Japan; b Department of Surgery, Kimitsu Chuo Hospital, 1010 Sakurai, Kisarazu, Chiba 292-8535, Japan; c Department of Gastroenterology, Kimitsu Chuo Hospital, 1010 Sakurai, Kisarazu, Chiba 292-8535, Japan

**Keywords:** Hemorrhage, Serous cystic neoplasm, Pancreas, Therapeutic embolization, Pancreatectomy

## Abstract

**Introduction:**

Pancreatic serous cystic neoplasm (SCN) is usually benign and is often managed using imaging surveillance if asymptomatic. It has a higher incidence in females but is rare in younger age groups. Acute hemorrhagic complications associated with SCN are infrequent. Whether asymptomatic SCN can cause acute hemorrhage, especially in women of childbearing age, is not well-established.

**Presentation of case:**

A 30-year-old Japanese female, who was six months postpartum and under surveillance for asymptomatic pancreatic SCN, presented to the emergency department with gradually worsening left lateral abdominal pain. Regular ultrasound revealed no change in SCN size; however, no imaging surveillance had been conducted over the past two years. She had pain in the entire abdomen, which intensified around the navel and elicited guarding. Abdominal contrast-enhanced computed tomography revealed a cystic mass in the pancreatic tail with a contrast blush within the cyst and an adjacent retroperitoneal hematoma. Endovascular embolization was performed to control the hemorrhage. The patient had an uneventful medical recovery and was discharged five days after embolization. Five months after discharge, she underwent laparoscopic distal pancreatectomy and splenectomy as an elective surgery and was discharged uneventfully.

**Discussion:**

Even with periodic imaging surveillance, pancreatic SCN can suddenly cause spontaneous hemorrhage. Clinicians should be aware that pancreatic SCN can potentially cause life-threatening complications, including spontaneous hemorrhage.

**Conclusion:**

We report a case of an unexpected complication with spontaneous hemorrhage in a young woman who was under imaging surveillance for pancreatic SCN. The patient was successfully treated with angioembolization and planned laparoscopic surgery.

## Highlights

• Pancreatic serous cystic neoplasm (SCN) can cause rare spontaneous hemorrhage, even in younger patients.
• Spontaneous hemorrhage of pancreatic SCN can be life threatening.
• The effect of pregnancy on pancreatic SCN has not been elucidated but warrants attention.

## 1 Introduction

Pancreatic serous cystic neoplasm (SCN) constitutes 10–15 % of all cystic masses of the pancreas and is usually benign [1,2]. It is usually managed using imaging surveillance for asymptomatic cases. When present, the major symptoms are usually nonspecific and non-life-threatening, including abdominal pain and the presence of a palpable mass [3]. A few cases of pancreatic SCN complicated by spontaneous hemorrhage have been reported, all of them in patients aged >70 years [4,5]. However, whether asymptomatic SCN can cause acute hemorrhage, especially in women of childbearing age, is not well-established. We present a case of spontaneous hemorrhage in a young woman who was under imaging surveillance for pancreatic SCN. This case report adheres to the SCARE 2020 criteria [6].

## 2 Presentation of case

A 30-year-old Japanese female, who was six months postpartum and under surveillance for macrocystic-type pancreatic SCN, presented to the emergency department with gradually worsening left lateral abdominal pain without any trauma episode. The SCN had previously been monitored by ultrasound for three to six months, showing no significant changes in size. However, owing to the patient's relocation, imaging surveillance had not been conducted for the past two years. The patient had no medical conditions aside from SCN and was not on anticoagulant or antiplatelet therapy.

Upon arrival, her blood pressure measured 121/77 mmHg, pulse rate 92 beats/min, and oxygen saturation 97 % on room air. Physical examination revealed tenderness in the entire abdomen, which intensified around the umbilicus and elicited guarding. Laboratory test results showed a hemoglobin level of 10.2 g/dL [normal range 11.2–14.8 g/dL]; however, coagulopathy was not confirmed (fibrinogen, 174 mg/dL [normal range 200–400 mg/dL]; platelet count, 323,000/μL [normal range 174,000–385,000/μL]; prothrombin time, 14.6 s [control time 12.2 s]; and activated partial thromboplastin time, 29.0 s [normal range 25–38 s]). Her white blood cell count was 26.0×10^9^/L [normal range 3.9×10^9^–9.4×10^9^/L]; C-reactive protein level, < 0.3 mg/dL (undetectable); and amylase level, estimated at 608 IU/L [normal range 25–120 IU/L].

**Fig. 1:**
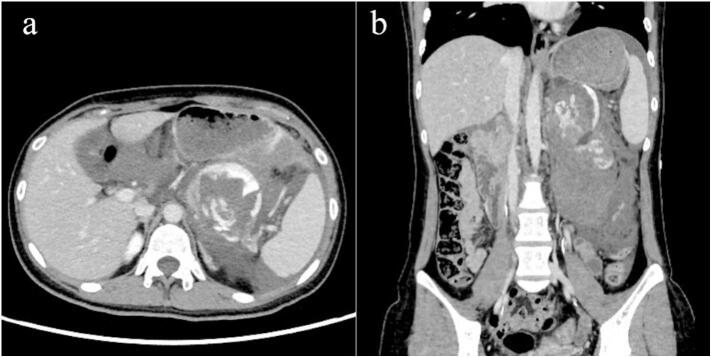
(a) Contrast-enhanced computed tomography (CT) showing contrast blush in the cyst, which delineates the margin of the cyst. (b) Coronal CT showing a retroperitoneal hematoma spreading caudally with concurrent flow into the retroperitoneum, in addition to an intra-cystic hemorrhage.

**Fig. 2:**
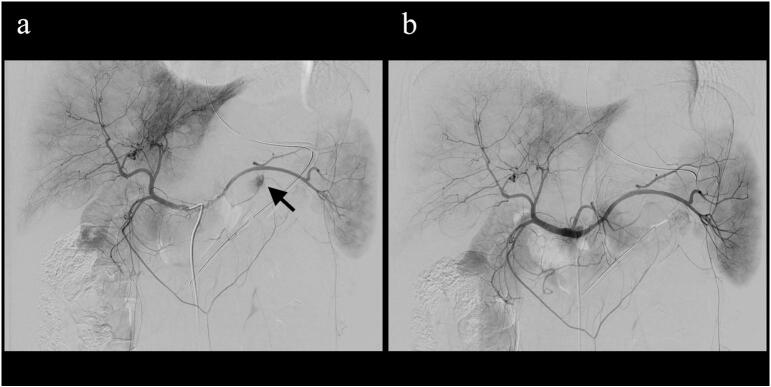
(a) Celiac artery angiography shows the contrast extravasation (arrow) into the cyst. (b) Celiac artery angiography after embolization. Embolization was successfully performed using *N*-butyl cyanoacrylate. No contrast leakage is observed.

**Fig. 3:**
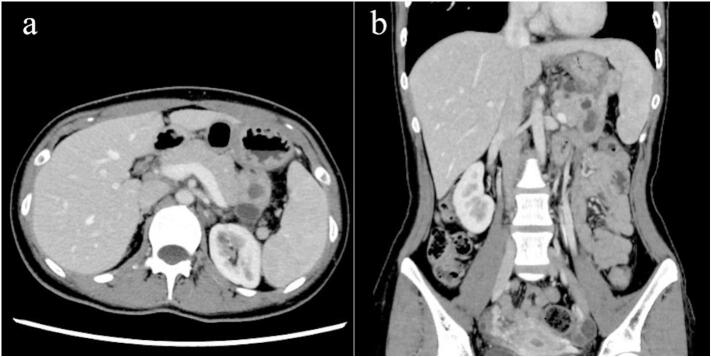
(a) Contrast-enhanced computed tomography (CT) at three months after discharge reveals that the peripancreatic hematoma is remarkably reduced. CT shows a cystic lesion in the tail of the pancreas.

Abdominal contrast-enhanced computed tomography (CT) revealed a cystic mass in the pancreatic tail with a contrast blush within the cyst and an adjacent retroperitoneal hematoma (Fig. 1a, b). Endovascular embolization was performed to control the hemorrhage under general anesthesia because the patient was unable to rest owing to pain and had become lethargic. Celiac artery angiography showed extravasation of the contrast medium into the cyst (Fig. 2a); the microcatheter was directed to the hemorrhage site and embolized using 33 % *N*-butyl cyanoacrylate and iodized oil mixture. The patient required four units of packed red blood cells and six units of fresh frozen plasma transfusion during angioembolization. Postembolization contrast leakage was not observed (Fig. 2b); the patient had an uneventful medical recovery and was discharged five days after embolization. In the outpatient clinic, contrast-enhanced CT performed three months later revealed that the peripancreatic hematoma was remarkably reduced (Fig. 3a, b).

To avoid the risk of re-bleeding and ensure resolution of the hematoma, the patient underwent laparoscopic distal pancreatectomy and splenectomy as elective surgery for pancreatic SCN at five months post-discharge. Pathology results from the specimen revealed multiple cystic structures lined with cuboidal-to-low-columnar epithelial cells, indicating serous cystadenoma. The patient was safely discharged after laparoscopic surgery. Finally, the patient was diagnosed with spontaneous intracapsular hemorrhage associated with pancreatic SCN.

## 3 Discussion

This report shows the successful management of a pancreatic SCN with spontaneous hemorrhage in a young woman under imaging surveillance with emergency endovascular embolization and planned laparoscopic surgery. Pancreatic SCN is mostly benign and rarely becomes symptomatic [1,2,4]. It is often diagnosed in individuals aged 50–70 years, with a higher incidence in women than in men [1,7]. Nevertheless, owing to the escalating utilization of superior cross-sectional imaging techniques and the growing inclination of healthy individuals to undergo proactive healthcare screenings, including diagnostic imaging procedures, pancreatic cystic neoplasms are increasingly being identified [1]. Consequently, the possibility of incidentally discovering SCN among young individuals may also increase. Imaging surveillance is typically chosen for asymptomatic patients; opting for resection remains controversial [8]. Therefore, surveillance must consider potential malignancies and mechanical complications associated with tumor growth [9]. According to current guidelines, asymptomatic patients with radiological evidence of SCN should be followed-up for one year. After one year, symptom-based follow-up is recommended [10]. Although the threat of hemorrhage for individuals with SCN has not significantly influenced treatment or surveillance algorithms to date, it is crucial to know that it can occur.

Spontaneous hemorrhaging associated with SCN is rare; however, it can be fatal. Only a few cases of hemorrhage from pancreatic SCN have been reported in the literature [4,5]; this case is unique because such hemorrhages are more commonly reported in older individuals. Mucinous cystic neoplasm can occur in the pancreas, and an association between this type of tumor and pregnancy has been reported in the literature, supporting the consideration of surgical resection when the tumor is showing rapid growth and is at risk of spontaneous rupture [11]. The observed growth of mucinous cystic neoplasms during pregnancy suggests a hormonal influence on tumor biology. However, the mechanism underlying this influence is not yet fully understood [12]. Additionally, it remains unknown whether this phenomenon extends to SCN. While the association between SCN and pregnancy remains unclear, this single case report highlights the need to consider the possibility of hemorrhage in SCN postpartum. If patients under imaging surveillance for SCN become pregnant, heightened caution may be warranted.

## 4 Conclusion

We reported a case of spontaneous hemorrhage of an asymptomatic pancreatic SCN in a young woman. The patient was successfully treated with angioembolization and planned laparoscopic surgery. Despite the implementation of regular imaging surveillance, it is crucial for clinicians to be fully aware that pancreatic SCN, even in asymptomatic young patients, carries the potential for serious and life-threatening complications, including spontaneous hemorrhage.

## Consent for publication

Written informed consent was obtained from the patient for publication and any accompanying images. A copy of the written consent is available for review by the Editor-in-Chief of this journal on request.

## Ethical approval

The requirement for ethical approval was waived by our institution. The patient provided informed consent for the publication of medical information in this case report.

## Funding

This study did not receive any specific grants from funding agencies in the public, commercial, or not-for-profit sectors.

## Author contribution

ToS contributed to the study's conception and design. ToS, AO, and YY contributed to patient care. The first draft of the manuscript was written by ToS, and all authors commented on previous versions of the manuscript. All authors read and approved the final manuscript.

## Guarantor

Toshinao Suzuki.

## Research registration number

Not applicable.

## Declaration of competing interest

The authors declare that they have no competing interests.

## Data Availability

Not applicable.

## References

[1] van Huijgevoort N.C.M., Del Chiaro M., Wolfgang C.L., van Hooft J.E., Besselink M.G. (2019). Diagnosis and management of pancreatic cystic neoplasms: current evidence and guidelines. Nat. Rev. Gastroenterol. Hepatol..

[2] Huh J., Byun J.H., Hong S.M., Kim K.W., Kim J.H., Lee S.S. (2016). Malignant pancreatic serous cystic neoplasms: systematic review with a new case. BMC Gastroenterol..

[3] Pointer L., Rothermel L.D., Strosberg C., Anaya D., Hodul P. (2019). Giant symptomatic serous cystadenoma mimicking carcinoma: a case report and literature review. Int. J. Surg. Case Rep..

[4] Amaral M.J., Serôdio M., Ramalhosa F., Tralhão J.G. (2020). Pancreatic microcystic serous cystadenoma: a lethal disease? Rare case of a life-threatening haemorrhage. BMJ Case Rep..

[5] Tamura S., Yamamoto Y., Okamura Y., Sugiura T., Ito T., Ashida R. (2018). A case of duodenal hemorrhage due to arteriovenous malformation around a serous cystic neoplasm. Surg. Case Rep..

[6] Sohrabi C., Mathew G., Maria N., Kerwan A., Franchi T., Agha R.A. (2023). The SCARE 2023 guideline: updating consensus Surgical CAse REport (SCARE) guidelines. Int. J. Surg. Lond. Engl..

[7] Scholten L., van Huijgevoort N.C.M., van Hooft J.E., Besselink M.G., Del Chiaro M. (2018). Pancreatic cystic neoplasms: different types, different management, new guidelines. Visc. Med..

[8] Lee L.S. (2021). Updates in diagnosis and management of pancreatic cysts. World J. Gastroenterol..

[9] Hwang H.K., Kim H., Kang C.M., Lee W.J. (2012). Serous cyst adenoma of the pancreas: appraisal of active surgical strategy before it causes problems. Surg. Endosc..

[10] European Study Group on Cystic Tumours of the Pancreas (2018). European evidence-based guidelines on pancreatic cystic neoplasms. Gut.

[11] Revoredo F., de Vinatea J., Reaño G., Villanueva L., Kometter F., Arenas J. (2020). Mucinous cystic neoplasms of the pancreas associated with pregnancy: two case reports. Medicine (Baltimore).

[12] Kleeff J., Holzapfel K., Steiger K., Esposito I. (2015). Progression of a cystic pancreatic lesion during pregnancy. J. Pancreas.

